# Unprecedented rise of monkeypox in Europe and America: Are Asian countries ready for a new outbreak during the ongoing COVID-19 pandemic?

**DOI:** 10.7189/jogh.12.03066

**Published:** 2022-08-31

**Authors:** Sakirul Khan, Sheikh Mohammad Fazle Akbar, Takaaki Yahiro, Mamun Al Mahtab, Kazunori Kimitsuki, Akira Nishizono

**Affiliations:** 1 Department of Microbiology, Faculty of Medicine, Oita University, Yufu, Oita, Japan; 2 Department of Gastroenterology and Metabology, Ehime University Graduate School of Medicine, Toon, Ehime, Japan; 3 Research Center for Global and Local Infectious Diseases, Faculty of Medicine, Oita University, Yufu, Oita, Japan; 4 Division of Interventional Hepatology, Bangabandhu Sheikh Mujib Medical University, Dhaka, Bangladesh

The monkeypox virus is a large DNA virus and the etiological agent of a zoonotic disease known as monkeypox (MPX). It has an incubation period of 5-21 days and the symptoms range from headache, muscle aches, back pain, swollen lymph nodes, and fever to respiratory distress, many of them are similar to symptoms of the ongoing pandemic of coronavirus 2019 (COVID-19). The point of interest, as well as concern, lies in the fact that, although MPX has been endemic in west and central Africa since 1970 [1], the present outbreak has occurred in non-endemic regions [2,3]. This is not an exceptional event as MPX outbreaks have been found in the USA in 2003 and the UK, Israel, and Singapore in 2017, with most cases associated with travellers returning from endemic countries, or due to nosocomial contact or contact with infected imported rodents [4,5]. However, the magnitude of previous infections is not comparable with the malignant upsurge in 2022. In fact, the current outbreak of MPX does not seem to be a continuation of the previous ones, because person-to-person transmissions have been reported in at least 88 countries or territories within two months (as of August 5th, 2022) and the list of new countries is growing on a regular basis [6]. Such an unexpected increase in MPX cases in non-endemic countries is raising concerns about a new pandemic threat. Moreover, as human-to-human transmission occurs through close contact with infected persons (eg, respiratory droplets, skin-on-skin, sexual contact, or fomites), detecting and treating MPX is significantly more complicated. This situation induced the World Health Organization (WHO) to declare the current outbreak of MPX a public health emergency of international concern (PHEIC) – the highest level of alert [7]. Besides the effects of the ongoing COVID-19 pandemic, the present realities of the Russia-Ukraine conflict as well as emergency situations in the Taiwan straits demand highly comprehensive and concerted efforts to tackle the spread of MPX in Asia, which represents about 60% of the total world population. This has become more relevant as China has stopped many collaborations, including those of scientific nature, with the USA [8].

Photo: People with monkeypox get papular lesions in the hand and leg. Photo from WHO homepage. Source: https://www.who.int/health-topics/monkeypox#tab=tab_1.

**Figure 1:**
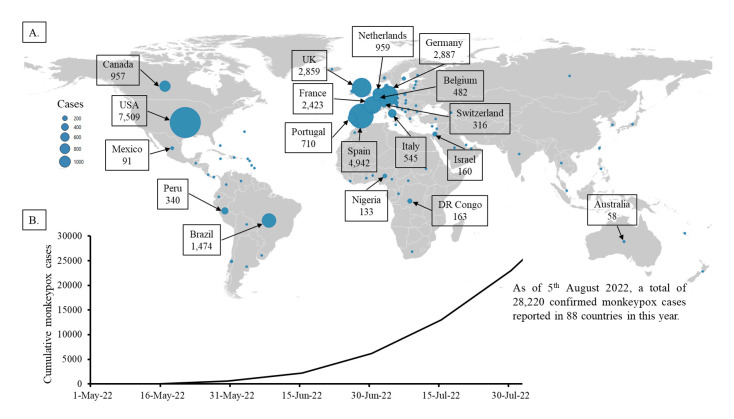
Current global monkeypox (MPX) infection patterns as of August 5, 2022. Panel A: Geographical distribution of confirmed MPX cases. Bubbles indicate the confirmed MPX cases by representative country. Map was created using QGIS software version 3.26.1 (www.qgis.org) and Microsoft^®^ Excel. Panel B: Cumulative number of confirmed MPX cases worldwide.

The recent emergence of this zoonotic disease worldwide has generated a great concern to update the information on MPX outbreaks as the world passes through a pandemic situation. There is a need for more research in the epidemiology, ecology, and biology of the virus in endemic areas to better understand and prevent human infections. In this communication, we have analysed the prevalence of current MPX infection and addressed some concerns about the spread of this zoonotic disease in developing and resource-constrained countries in Asia and countries with similar situations in other regions. The present infection patterns indicate that the majority of MPX cases are occurring in Europe and North and South America (**Figure 1**, Panel A). Outside of historically MPX endemic countries (Africa), the first case of the current outbreak of MPX was reported in Europe on May 6, 2022. Since then, MPX has rapidly transmitted to other continents, with rising tolls. In fact, North America (USA) reported its first MPX case on May 18, followed by Australia on May 20, Asia (Israel) on May 21, and South America (Brazil) on June 8. As of August 5, 2022, a total of 28 220 confirmed MPX cases have been reported in 88 countries (**Figure 1**). Moreover, 1685 suspected cases have been reported since May 2022 [9]. Out of confirmed cases, about 6000 MPX-positive patients have been detected from May to June 2022. However, there has been a tremendous jump in newly diagnosed cases of MPX during July 2022 (**Figure 1**, Panel B) with about 17 000 reported cases of MPX; the disease’s prevalence may have dramatically risen again in August 2022, as more than 5000 MPX cases have already been reported within the first five days. In the current outbreak, at least three countries (Brazil, Spain, and India) outside of the historically endemic region have reported deaths from MPX. The pattern of present MPX cases indicates that the scenario may worsen in the coming days, as MPX cases are rising in many new countries regularly [6].

Among the confirmed MPX cases, only 345 have been reported in seven African countries that historically have MPX. On the other hand, 27 875 cases are reported in countries outside of the endemic areas. However, most of the cases are reported in Europe and America. In fact, 14 countries in Europe and North and South America (Spain, Germany, UK, France, The Netherlands, Portugal, Italy, Belgium, Switzerland, USA, Canada, Mexico, Brazil, and Peru) reported more than 90% of confirmed MPX cases (**Figure 1**, Panel A). On the other hand, except for Israel (160 cases), only a few countries in Asia including South East and Middle East region reported a small number of MPX cases (16 cases in the United Arab Emirate, 15 in Singapore, six in India, five in Saudi Arabia, four in Thailand, two in Qatar, two in Taiwan, two in Japan, one in South Korea, and one in the Philippines). Similarly, except for Australia (58 cases), only two countries in the Oceania region reported a small number of MPX cases (three cases in New Zealand and one in New Caledonia).

In most reported cases in Europe and America, patients with MPX had no travel history to the endemic areas of Africa but were diagnosed through primary care and sexual health services. In fact, although there is no prior evidence of sexual transmission of MPX, the recent cases are mainly reported in homosexual, bisexual, or other men who have sex with men [10,11]. The MPX virus was also reportedly found in the semen of infected patients [12]. It is, therefore, possible that MPX may spread by close contact during sexual activity. Due to social and religious matters, many Asian countries have not granted equal rights to the LGBTQI+ community. Whether such regulations can prevent the spread of MPX infection in this region remains questionable. With the experience of the ongoing pandemic, only a few countries in Asia (mostly in China) were affected by COVID-19 in the early pandemic time when the Wuhan and/or Alpha variant of severe acute respiratory syndrome coronavirus 2 (SARS-CoV-2) devastated Europe and America. Later, it distorted the health care delivery system of Asia after the emergence of the mutant SARS-CoV-2 delta variant in India, the only South Asian country currently reporting MPX cases and MPX-related death. So far, no new variant of the MPX virus has been discovered. However, we cannot exclude the possibility that a more virulent type of MPX virus could develop in near future. In fact, pieces of evidence suggested that the MPX virus has developed an average of 50 single-nucleotide polymorphisms [13]. The MPX virus could possibly become more infectious and more adapted to spread among humans. Several research groups have already projected the future incidence of MPX worldwide and in specific regions, predicting many more cases by the end of 2022 [14]. These facts indicate that the MPX virus may spread more vigorously in the highly populated countries in the Asian region in near future.

New therapeutics and vaccines offer hope for the treatment and prevention of MPX; however, more research must be done before they are ready to be deployed in an endemic setting. One of the major fighting tools in our hands is the use of the available smallpox vaccine that provides cross-immunity to the MPX virus, as severe complications and sequelae were found to be more common among unvaccinated individuals than vaccinated patients [15]. Therefore, the vaccination can be accomplished for high-risk populations and post-exposure prophylaxis. However, developing and resource-constrained countries neither have smallpox vaccines nor they are able to manufacture it quickly. Moreover, the spread of the virus would not be binding to any rule. The mistake of COVID-19 management should not be repeated as the world is again on the brink of another pandemic of MPX. Therefore, developing and resource-constrained countries, particularly in the Asian region, where the health care system is already under pressure due to the COVID-19 pandemic and has limited access to testing resources and vaccines for MPX, need urgent preparation to tackle the outbreak of MPX.

The current outbreak of MPX in non-endemic regions has shown a yellow card for still less affected countries and highlights how negligence has allowed the virus to spread from Africa to other parts of the world. It should also serve as a reminder that in an inter-connected and globalized world, no region or country is safe from zoonotic pathogens like the MPX virus unless proper preventive measures have been taken quickly. Therefore, considering the severeness of the co-existence of a pandemic (COVID-19) and an epidemic (MPX), developing a comprehensive and evidence-based scientific program at all levels is essential in controlling the spread of MPX infections.
